# Clinical characteristics and outcome of primary brain abscess: a retrospective analysis

**DOI:** 10.1186/s12879-021-06947-2

**Published:** 2021-12-13

**Authors:** Junying Huang, Haining Wu, Honghong Huang, Weiqi Wu, Bowen Wu, Lingxing Wang

**Affiliations:** Department of Neurology, the Second Affiliated Hospital of Fujian Medical University, Quanzhou, 362000 Fujian China

**Keywords:** Comorbidities, Headache, Primary brain abscess, Patient outcome, Risk factor

## Abstract

**Background:**

Patients with primary brain abscess often present with atypical symptoms, and the outcome varies. We investigated the demographic, laboratory, and neuroimaging features of patients with brain abscess at our hospital and identified factors associated with their outcomes.

**Methods:**

We retrospectively collected the data of patients diagnosed with primary brain abscess at our hospital between January 2011 and December 2020. Their clinical characteristics, predisposing factors, laboratory and neuroimaging findings, treatment, and outcome were analyzed.

**Results:**

Of the 57 patients diagnosed with primary abscess, 51 (89.47%) were older than 40 years, and 42 (73.68%) were male. Only eight patients (14.04%) showed the classical triad of headache, fever, and focal neurological deficit. Fifteen patients (26.31%) had comorbidities, of which diabetes mellitus was the most common. Positive intracranial purulent material cultures were obtained in 46.15% of the patients, and gram-negative enteric bacteria were found in 33.33% of them, with *Klebsiella pneumoniae* being the most frequently observed. Surgical treatment, most commonly in the form of stereotactic drainage, was received by 54.39% of the patients. Good outcomes were achieved in 75.44% of the patients. Multivariate logistic regression analysis showed that patients with headaches were more likely to have a poor outcome (odds ratio 6.010, 95% confidence interval 1.114–32.407, p = 0.037).

**Conclusions:**

Male patients and those older than 40 years were more susceptible to brain abscess than female patients and those younger than 40 years, respectively. Only a few patients showed the classical triad of clinical symptoms. Diabetes mellitus was the most common comorbidity. Positive intracranial specimens’ culture results were uncommon, with gram-negative enteric bacteria, especially *Klebsiella pneumoniae*, being the main organisms found. Most patients had a good outcome, and the presence of headache may influence the outcome.

## Background

Brain abscess is a focal intraparenchymal infection characterized by an encapsulated collection of purulent material, immune cells, and other materials following bacterial or fungal infection. It has a prevalence of approximately 0.3–1.3 per ten thousand people per year [1], with a high incidence in developing countries (8%) [2]. It is potentially life-threatening and is often associated with poor outcomes or persistent neurological sequelae. Secondary brain abscess results from open brain injury or brain surgery, whereas primary brain abscess occurs due to hematogenous or contiguous spread of infection and is not caused by any surgical procedure [3–5]. Most patients with primary brain abscess present with atypical symptoms, which renders diagnosis challenging. Here, we retrospectively collected and analyzed the information of patients diagnosed with primary brain abscess at our hospital in the last ten years to determine the predisposing risk factors, clinical characteristics, and predictors of outcome.

## Methods

### Inclusion and exclusion criteria

We retrospectively retrieved the electronic medical records of patients diagnosed with primary brain abscess at our hospital between January 2011 and December 2020. Patients who met at least one of the following criteria were included: (1) evidence of infection in brain specimens obtained through surgical aspiration or lesion excision; (2) brain magnetic resonance imaging and/or computed tomography findings of brain abscess and reversal of brain lesion with antibiotic therapy. Patients with secondary brain abscess and those with intracranial empyema, including subdural and epidural abscess, were excluded. This study was approved by the ethics committee of the Second Affiliated Hospital of Fujian Medical University (NO: 2020-372).

### Clinical and neuroimaging data

Data regarding the patients’ demographic characteristics, predisposing factors, clinical characteristics, symptom duration, neurological status at admission, blood and cerebrospinal fluid (CSF) examination results, culture results of blood, CSF or brain abscess purulent material (including bacteria and fungus; if there were polymicrobial infection, all causative organisms were included), antibiotic treatment, type of surgery, number of abscesses, and location of the brain abscess on magnetic resonance imaging or computed tomography were extracted from electronic medical records. The brain abscess volume (in mm^3^) was calculated using the formula 0.5 × X × Y × Z [6], where X, Y, and Z were the largest diameters of the abscess in the X-, Y-, and Z-axes, respectively. If there were multiple brain abscesses, the largest abscess was measured.

### Outcome assessment

The outcome was evaluated based on the Glasgow Outcome Scale score at discharge (1, death; 2, persistent vegetative state; 3, severe disability; 4, moderate disability; 5, good recovery). A score of 1–3 was regarded as a poor outcome, and a score of 4–5 was regarded as a good outcome, which indicated the patient was independent in daily life.

### Statistical analysis

Statistical analyses were performed using SPSS 22.0. Continuous data were expressed as means ± standard deviations or medians and analyzed using the independent t-test or Mann–Whitney U test. Categorical data were analyzed using the chi-square test. To identify factors affecting the outcome, we performed multivariate logistic regression analysis of variables with p < 0.2 in the univariate analysis. The level of significance was set at p < 0.05.

## Results

### Clinical characteristics

Fifty-seven patients were diagnosed with primary brain abscess during the study period. The mean age was 52.26 ± 14.09 years (range 6–80). Six patients (10.53%) were < 40 years old, 32 (56.14%) were 40–60 years old, and 19 (33.33%) were > 60 years old. Forty-two patients (73.68%) were male, and 15 patients (26.32%) were female. The mean duration of hospitalization was 28.61 ± 15.18 days, and the median symptom duration at admission was six days. The patients’ symptoms at admission included headache (52.63%), fever (45.61%), hemiplegia (45.61%), confusion (31.68%), nausea and vomiting (22.81%), epilepsy (14.04%), aphasia (12.28%), and neck stiffness (5.26%).

### Predisposing factors and comorbidities

The patients’ predisposing factors included adjacent site infection such as paranasal sinusitis (seven patients, 12.28%), chronic otitis media or mastoiditis (five patients, 8.77%), and dental infection (one patient, 1.75%); hematogenous infection such as pneumonia (nine patients, 15.79%) and bacterial endocarditis (one patient, 1.75%); and cyanotic heart disease (one patient, 1.75%). The patients’ comorbidities included immunocompromising diseases such as diabetes mellitus (nine patients, 15.78%), tumor (three patients, 5.26%), liver cirrhosis (one patient, 1.75%), stroke (two patients, 3.50%) and granulocytopenia (one patient, 1.75%), and one patient experienced both diabetes mellitus and stroke. Moreover, three patients (5.26%) previously underwent head or neck surgery, with one undergoing surgery for intracranial hematoma approximately five years ago, one undergoing surgery for subdural hematoma one year ago, and one for cervical disc herniation three years prior.

### Results of blood, cerebrospinal fluid, and brain abscess purulent material culture

Thirty patients (53%) had elevated white blood cell counts (> 1000 × 10^6^/L; Table 1) on peripheral blood testing. Blood culture was performed in 25 patients (43.86%), and positive results were obtained in four (16.0%) of them; bacteria of the streptococcus species were isolated in three patients (75%; Table 2). Lumbar puncture was performed in 18 patients (31.58%; Table 1), and an increased cell count (> 5 × 10^6^/L) was observed in the CSF of 17 patients (94.44%), with two patients showing cell counts of > 1000 × 10^6^/L. Moreover, 17 patients (94.44%) showed CSF protein elevation (> 0.45 g/L). CSF culture was performed in 16 patients (28.07%), and positive results were obtained in four of them (25%), with *Streptococcus* being the most commonly isolated organism (two patients, 50%; Table 2). Culture of the intracerebral specimens obtained during surgery was performed in 26 patients (45.61%), and positive results were obtained in 12 of them (46.15%; Table 2). In the identified organism, Gram-negative enteric bacteria, including *Proteus mirabilis* and *Klebsiella pneumoniae*, were observed in four patients (33.33%), and staphylococcal and fungal infections were observed in two patients each.

**Table 1:** Results of blood and cerebrospinal fluid in patients with primary brain abscess

Variables	n/N (%)
Blood test	
Increased WBC	30/57 (53.00)
Blood culture	25/57 (43.86)
Positive blood culture	4/25 (16.00)
CSF test	
LP	18/57 (31.58)
Pleocytosis	17/18 (94.44)
Elevated CSF protein	17/18 (94.44)
Culture	16/57 (28.07)
Positive CSF culture	4/16 (25.00)

*CSF* cerebrospinal fluid; *LP* lumbar puncture; *WBC* white blood cell

**Table 2:** Organisms isolated from primary brain abscess culture specimens

Case number	Sex	Age	Culture specimen	Organism
1	M	56	Intracranial purulent material	*Sphingomonas paucimobilis*
2	M	43	Intracranial purulent material	*Staphylococcus aureus*
3	M	41	Intracranial purulent material	*Proteus mirabilis*
4	M	43	Intracranial purulent material	*Rhizopus oryzae*
5	M	65	Intracranial purulent material	*Klebsiella pneumoniae*
6	M	45	Blood	*Streptococcus anginosus*
7	M	57	Intracranial purulent material	*Streptococcus anginosus*
8	M	38	Intracranial purulent material	*Prevotella*
9	M	59	CSF	*Neisseria cinerea*
10	M	20	Blood + CSF	*Streptococcus pneumoniae*
11	F	60	CSF	*Klebsiella pneumoniae*
12	M	54	Blood + intracranial purulent material	*Enterococcus faecalis*
13	M	60	Intracranial purulent material	*Staphylococcus aureus*
14	M	60	Intracranial purulent material	*Klebsiella pneumoniae*
15	M	52	Intracranial purulent material	*Klebsiella pneumoniae*
16	M	33	CSF	*Streptococcus intermedius*
17	F	48	Blood	*Viridans streptococci*
18	M	38	Intracranial purulent material	*Saccharomyces albicans*

*CSF* cerebrospinal fluid

### Neuroimaging findings

All the patients underwent at least one neuroimaging study. Forty-nine patients (85.96%) underwent enhanced brain magnetic resonance imaging, ten (17.54%) underwent enhanced brain computed tomography, and four (0.02%) underwent both. Forty patients (70.17%) had a single abscess, and 17 patients (29.83%) had multiple abscesses. The abscess was located in the frontal lobe in 28 patients (49.12%), temporal lobe in 16 patients (28.07%), parietal lobe in 16 patients (28.07%), occipital lobe in 11 patients (19.30%), basal ganglia in four patients (7.02%), cerebellum in nine patients (15.79%), and brainstem in two patients (3.51%). The median abscess volume was 9.40 cm^3^ (95% confidence interval 9.68–17.48), with small (< 1 cm^3^), medium (1–10 cm^3^), and large (> 10 cm^3^) lesions in seven (12.28%), 25 (43.86%), and 25 (43.86%) patients, respectively.

### Treatment and outcome

Fifty-five patients (96.49%) received antibiotic therapy after admission, and two patients (3.51%) received antibiotic therapy after diagnosis with neuroimaging or surgery. Initial empirical antibiotic therapy included ceftriaxone, metronidazole, piperacillin-tazobactam, vancomycin, or a combination of these drugs, and it was adjusted according to the results of sensitivity testing. The mean duration of antibiotic therapy was 28.16 ± 15.18 days. Thirty-one patients (54.39%) underwent surgery, of whom 23 (74.19%) and eight (25.81%) underwent stereotactic drainage and craniotomy, respectively. Table 3 shows the comparison of patients with and without surgery, and significant differences in the volume of the abscess (p = 0.001) and some symptoms at admission, including hemiplegia (p = 0.037) or aphasia (p = 0.039), were observed between the two groups. At discharge, 14 patients (24.56%) had a poor outcome, including two (3.50%) who died, and the remaining 43 (75.44%) had a good outcome. Multivariate analysis was performed to identify factors associated with a poor outcome. The following variables were tested: headache, confusion, age, adjacent site infection, and type of surgery. The presence of headache was independently associated with a poor outcome (odds ratio 6.010, 95% confidence interval 1.114–32.407, p = 0.037; Table 4).

**Table 3:** Comparison of patients with or without surgery

	Surgery (n = 31)	No surgery (n = 26)	Total (n = 57)	X^2^	P
Age, years, n (%)					
< 40	4 (66.70)	2 (33.30)	6	3.496	0.192
40–60	20 (62.50)	12 (37.50)	32		
≥ 60	7 (36.80)	12 (63.20)	19		
Male n (%)	25(80.60)	17(65.40)	42	1.700	0.191
Comorbidities, n (%)	6 (40.00)	9 (60.00)	15	1.698	0.193
Headache, n (%)	19 (61.30)	12 (38.70)	31	2.044	0.153
Fever, n (%)	12 (46.20)	14 (53.80)	26	1.306	0.253
Confusion, n (%)	12 (66.70)	6 (33.30)	18	1.599	0.164
Hemiplesia, n (%)	18 (69.20)	8 (30.80)	26	4.427	0.037*
Epilepsy	5 (62.50)	3 (37.50)	8	–	0.715
Aphasia	1 (14.30)	6 (85.70)	7	–	0.039*
Raised WBC	15 (50.00)	15 (50.00)	30	0.491	0.483
Number of abscess					
Single	24 (60.00)	16 (40.00)	40	1.704	0.192
Multiple	7 (41.20)	10 (58.80)	17		
Location of abscess, n (%)					
Basal ganglia	1 (25)	3 (75)	4	7.442	0.266
Frontal lobe	15 (53.6)	13 (46.4)	28		
Temporal lobe	6 (37.5)	10 (62.5)	16		
Parietal lobe	10 (62.5)	6 (37.5)	16		
Occipital lobe	2 (18.2)	9 (81.8)	11		
Cerebellum	5 (55.6)	4 (44.4)	9		
Brainstem	1 (50.0)	1 (50.0)	2		
Volume of abscess, n (%)					
< 1cm^3^	1 (14.3)	6 (85.7)	7	13.203	0.001*
1–10 cm^3^	10 (40.0)	15 (60.0)	25		
> 10cm^3^	20 (80.0)	5 (20.0)	25		

*p < 0.05 was considered statistically significant

**Table 4:** Risk factors for poor outcome in patients with primary brain abscess

	Good outcome (n = 43)	Poor outcome (n = 14)	Total (n = 57)	Univariate analysis		Logistic regression analysis		
				χ^2^	p	OR	p	95% CI
Age, years, n (%)								
< 40	3 (50.00)	3 (50.00)	6	2.305	0.328	0.365		
40–60	25 (78.13)	7 (21.87)	32					
> 60	15 (78.95)	4 (21.05)	19					
Male n (%)	31 (73.81)	11 (26.19)	42	0.017	0.898			
Symptom duration at admission* (days)	5.50	5.50		U = 264.50	0.575			
Duration of hospitalization* (days)	30.00	21.00		U = 231.00	0.194			
Adjacent site infection, n (%)	12 (92.31)	1 (7.69)	13	1.541	0.214	0.051		
Comorbidities, n (%)	11 (73.30)	4 (26.70)	15	0.049	0.825			
Headache, n (%)	26 (86.67)	4 (13.33)	30	4.309	0.038	0.037	6.010	1.114–32.407
Fever, n (%)	18 (69.23)	8 (30.77)	26	0.994	0.319			
Confusion, n (%)	12 (66.67)	6 (33.33)	18	0.510	0.475	0.164		
Hemiplegia, n (%)	18 (69.23)	8 (30.77)	26	0.994	0.319			
Epilepsy	5 (62.50)	3 (37.50)	8	0.841	0.359			
Aphasia	5 (71.43)	2 (28.57)	7	0.000	1.000			
Increased WBC count	22 (73.33)	8 (26.67)	30	0.151	0.697			
Number of abscesses								
Single	31 (77.50)	9 (22.50)	40	0.048	0.827			
Multiple	12 (70.59)	5 (29.41)	17					
Abscess location, n (%)								
Basal ganglia	1 (25)	3 (75)	4	6.82	0.313			
Frontal lobe	22 (78.6)	6 (21.4)	28					
Temporal lobe	12 (75)	4 (25)	16					
Parietal lobe	11 (68.8)	5 (31.2)	16					
Occipital lobe	6 (54.5)	5 (45.5)	11					
Cerebellum	7 (77.8)	2 (22.2)	9					
Brainstem	1 (50)	1 (50)	2					
Abscess volume, n (%)								
< 1 cm^3^	6 (85.7)	1 (14.3)	7	1.258	0.620			
1–10 cm^3^	20 (80.0)	5 (20.0)	25					
> 10 cm^3^	17 (68.0)	8 (32.0)	25					
Surgery, n (%)								
No	17 (65.38)	9 (34.62)	26	3.852	0.132	0.685		
Drainage	18 (78.26)	5 (21.74)	23					
Craniotomy	8 (100.00)	0	8					

*Median; *OR* odds ratio; *CI* confidence interval; *WBC* white blood cell. Multivariate logistic regression analysis was performed for factors with p < 0.20 in the univariate analysis. p < 0.05 was considered statistically significant

## Discussion

42.11% of the patients had some predisposing factors, and 22.81% (13 patients) had adjacent site infection, including paranasal sinusitis, chronic otitis media, mastoiditis, and dental infection. A previous study in a developing country reported otitis media as the most common source of intracranial suppuration [7]. However, this was not the case in our study, possibly due to improvements in the treatment of otitis media in recent decades. It is important to recognize predisposing factors because eliminating the underlying infection helps to avoid prolonged infection. Comorbidities, including diabetes mellitus, tumor, liver cirrhosis, history of stroke, and granulocytopenia, were noted in 26.31% of the patients in our study. Among these, a history of stroke was reported to be a common comorbidity as damaged brain tissues might be vulnerable to invasion by infectious organisms [8]. Other comorbidities, including human immunodeficiency virus infection, autoimmune disease, and immunosuppressive therapy, were reported in a previous study [9]. However, none of our patients had these comorbidities, and diabetes mellitus was the most common comorbidity observed in this study. The relationship between diabetes mellitus and susceptibility to infection has been reported [10]. Impaired glucose control may affect host defense and increase the risk of brain abscess.

The average age of the study participants was 52 years, and 56.14% of them were aged between 40 and 60 years. There are inconsistent reports regarding the age predilection of brain abscess. Some studies showed that individuals older than 40 years are more susceptible to brain abscess [4, 7, 11], whereas others revealed that brain abscess occurs more often in individuals younger than 40 years [12, 13]. The average age of participants in a meta-analysis conducted in 2014 was 33.6 years [1]. Therefore, the age group that is most affected is difficult to determine, and it may depend on the underlying predisposing factors for brain abscess. Moreover, we found that regardless of age, men were more susceptible to brain abscess than women at a ratio of 2.8:1. Other authors have reported similar findings [7, 14], although a female predilection was observed in one of these studies [7]. Headache, fever, and hemiplegia were the most common symptoms in our study, occurring in 52.63%, 45.61%, and 45.61% of the patients, respectively; however, this was consistent with previous reports [1, 11, 15]. Headache, fever, and focal neurological deficit are regarded as the classical symptoms of brain abscess. However, a few patients simultaneously experienced all three symptoms [1]. In our study, the classical triad of headache, fever, and hemiplegia was only observed in eight patients (14.04%); this was lower than the previously reported rate of 20% [1]. The presentations were insidious and atypical, and the absence of this classical clinical triad decreases the likelihood of brain abscess being suspected on initial examination.

Only half of the patients in this study had an elevated peripheral white blood cell count. Indicators of inflammation, such as white blood cell count, erythrocyte sedimentation rate, and C-reactive protein level, play a limited role in the diagnosis of brain abscess [1]. An increased cell count in the CSF, which indicates leptomeningeal affection, was observed in 94.44% of the patients who underwent lumbar puncture in this study, and this finding helped us determine the nature of the brain lesion in those patients. However, not all patients with brain abscess show leptomeningeal involvement, and the role of lumbar puncture in its diagnosis is limited. Moreover, lumbar puncture should be performed with caution. There are reports of clinical deterioration after lumbar puncture due to exacerbation of the brain tissue shift caused by the brain abscess, which may lead to death [1]. The rate of CSF culture positivity in our study was 25%, which is similar to the rate of 24% reported in a previous study [1]. Therefore, it is difficult to determine the causative organism solely based on the CSF culture result.

The rate of brain abscess purulent material culture positivity in our study was 46.15%, which is far lower than the previously reported rate of approximately 70% [1, 9]. This may be because we initiated antibiotic therapy before obtaining samples for culture, and the standard culture protocol followed at our hospital may result in certain organisms being undetected. The use of next-generation bacterial sequencing may help to identify more pathogens. In previous studies, the most commonly observed bacteria in patients with brain abscess were those of the streptococcus species [1]. However, in our study, gram-negative enteric bacteria especially *Klebsiella pneumonia* were the most common pathogens observed in intracranial specimens of brain abscess, and ceftriaxone, which is effective against gram-negative bacteria, was often initially administered as empirical antibiotic therapy. A previous study reported the frequent occurrence of *Klebsiella pneumoniae* in Asian patients [1]; it caused 10% of all brain abscesses in Taiwan. Moreover, we should also consider the possibility of the hypervirulent type in our cohort. Hypervirulent *Klebsiella pneumoniae*, which often occurs in immunocompetent individuals and leads to metastatic infection with poor prognosis, is being increasingly reported worldwide [16]. In addition, multiple brain abscesses tend to be caused by hypervirulent *Klebsiella pneumoniae*. The identification of hypervirulent *Klebsiella pneumoniae* is still difficult and depends on the combination of clinical features, a positive string test, and detection of hypervirulent genes [16]. In this study, patients with *Klebsiella pneumoniae* all had single brain abscess, and only one patient with diabetes had comorbid liver abscess; therefore, it is difficult to conclude without results of string test or gene detection. In the future, more attention should be paid to identifying the hypervirulent type of *Klebsiella pneumoniae.*

The most common brain abscess location in our study was the frontal lobe, followed by the temporal and parietal lobes, and most of the patients had single lesions. This result is consistent with a previous report [1, 7]. However, another study found that the temporoparietal region is the most common brain abscess location [17]. The location of a brain abscess partly depends on the route of infection transmission. Paranasal sinusitis is often associated with a frontal lobe abscess, and otitis media and mastoiditis are associated with temporal lobe or cerebellar abscesses.

Here, 54.39% of the patients underwent surgery. Most of them underwent stereotactic drainage, while others underwent craniotomy. The rate of surgical treatment was lower than that reported previously (60% to 87%) [1, 7, 11]; the reason for this phenomenon is unclear. A previous study showed that the number or size of abscesses or midline shifts may impact the decision for surgical therapy [8]. In our cohort, we observed a significant difference in the abscess volume between patients with and without surgery; however, no difference was observed in the abscess size. Moreover, significant differences in symptoms, including hemiplegia or aphasia, were similarly observed. It is possible that when patients have hemiplegia or aphasia, their family members are more prone to making surgical decisions to improve their quality of life, which helps to improve the acceptance of surgery. Nevertheless, further research is needed to determine the underlying reason for the low rate of surgical treatment. Although craniotomy was once thought to be associated with lower recurrence and mortality rates than stereotactic drainage [18], with the increasing availability of computed tomography, the difference between craniotomy and stereotactic drainage has become uncertain. A previous study found no difference in the effects, outcomes, and complications of these two surgical techniques [7]. Nevertheless, we should consider that good radiologic techniques cannot be substituted for the need for accurate microdata. Even after surgical treatment, long-term antibiotic therapy (4–8 weeks) was necessary. Moreover, 75.44% of the patients, who were independent in daily life, in our study had a good outcome, and only two patients died. The mortality rate, which was approximately 8–53% before 2014, has decreased in recent decades [19], and it was reported as 4.3% in 2018 [9]. The mortality rate in our study was lower than that in previous studies. We believe that this may reflect improvement in the treatment of brain abscess, although selection bias related to patient recruitment from our hospital, which is a tertiary hospital, should be considered. Previous studies have reported inconsistent findings regarding the factors associated with outcome. Landriel et al. [4] found that age, immunosuppression, and hematogenous spread were associated with a poor outcome. Zhang et al. [7] revealed that gender was associated with an unfavorable outcome. Another study [17] found that consciousness at presentation had prognostic value. Nevertheless, our findings indicate that headache, rather than confusion, age, adjacent site infection, or type of surgery, influenced outcomes. As a classical symptom of brain abscess, headache indicates possible intracranial hypertension, which may lead to a poor outcome.

This study has several limitations. First, the sample size was small. Second, it was a retrospective, single-center study. Consequently, our findings may not be generalizable to patients in different regions, and some data, including inflammatory marker levels, could not be collected and analyzed. Therefore, multicenter studies with larger samples should be conducted in the future.

## Conclusions

In conclusion, we retrospectively analyzed the data of 57 patients diagnosed with primary brain abscess over ten years. We found that men were more susceptible to primary brain abscess than women, and the most common comorbidity was diabetes mellitus. Gram-negative enteric bacteria, especially *Klebsiella pneumoniae*, were the most common pathogens. The presence of headache may be associated with a poor outcome.

## Data Availability

Data are available from the corresponding author upon reasonable request.

## Abbreviations

CSF: Cerebrospinal fluid
